# Recent Advances in Genome Editing Tools in Medical Mycology Research

**DOI:** 10.3390/jof7040257

**Published:** 2021-03-30

**Authors:** Sanaz Nargesi, Saeed Kaboli, Jose Thekkiniath, Somayeh Heidari, Fatemeh Keramati, Seyedmojtaba Seyedmousavi, Mohammad Taghi Hedayati

**Affiliations:** 1 Department of Medical Mycology, School of Medicine, Mazandaran University of Medical Sciences, Sari 481751665, Iran; nargesi.sanaz@gmail.com; 2 Department of Medical Biotechnology, School of Medicine, Zanjan University of Medical Sciences, Zanjan 4513956111, Iran; 3 Cancer Gene Therapy Research Center, Zanjan University of Medical Sciences, Zanjan 4513956111, Iran; 4 Fuller Laboratories, 1312 East Valencia Drive, Fullerton, CA 92831, USA; 5 Invasive Fungi Research Center, Communicable Diseases Institute, Mazandaran University of Medical Sciences, Sari 481751665, Iran; heidarisomaye@yahoo.com (S.H.); s.seyedmousavi@gmail.com (S.S.); 6 Department of Pathobiology, Faculty of Veterinary Medicine, Urmia University, Urmia 5756151818, Iran; fatemekeramati6464@gmail.com; 7 Clinical Center, Microbiology Service, Department of Laboratory Medicine, National Institutes of Health, Bethesda, MD 20892, USA

**Keywords:** gene editing techniques, CRISPR/Cas9, medically important fungi

## Abstract

Manipulating fungal genomes is an important tool to understand the function of target genes, pathobiology of fungal infections, virulence potential, and pathogenicity of medically important fungi, and to develop novel diagnostics and therapeutic targets. Here, we provide an overview of recent advances in genetic manipulation techniques used in the field of medical mycology. Fungi use several strategies to cope with stress and adapt themselves against environmental effectors. For instance, mutations in the 14 alpha-demethylase gene may result in azole resistance in *Aspergillus* *fumigatus* strains and shield them against fungicide’s effects. Over the past few decades, several genome editing methods have been introduced for genetic manipulations in pathogenic fungi. Application of restriction enzymes to target and cut a double-stranded DNA in a pre-defined sequence was the first technique used for cloning in *Aspergillus* and *Candida*. Genome editing technologies, including zinc-finger nucleases (ZFNs) and transcriptional activator-like effector nucleases (TALENs), have been also used to engineer a double-stranded DNA molecule. As a result, TALENs were considered more practical to identify single nucleotide polymorphisms. Recently, Class 2 type II Clustered Regularly Interspaced Short Palindromic Repeat (CRISPR)/Cas9 technology has emerged as a more useful tool for genome manipulation in fungal research.

## 1. Introduction

The fungi represent a large, diverse group of eukaryotic microorganisms that have a small genome size, short timeframes for growth and reproduction, and share homologous genes with humans [1]. *Aspergillus, Candida*, and *Cryptococcus* are the major fungal genera that cause opportunistic and life-threatening mycoses [2,3]. Over the past decades, genetic engineering has paved the way for the desired modifications by gene manipulation using a wide range of methods [4,5]. Genetic tools have been widely used to understand the virulence potential and pathobiology of fungal infections [6], as well as patterns of resistance development against antifungals [7]. Genome editing is a very useful tool which allows manipulation of a target site in a shorter period of time [8,9]. Here, we discuss the recent advances in genetic manipulation techniques that have been used in the field of medical mycology research with special emphasis on CRISPR/Cas9 technology. Additionally, we address the future perspectives of CRISPR/Cas9 applications in medically relevant fungi.

## 2. Genome Editing Technologies

Genome editingtechnology is a flexible engineering tool for genetic manipulation of microorganisms including fungi [8,9].

### 2.1. RNA Interference (RNAi)

**Figure 1:**
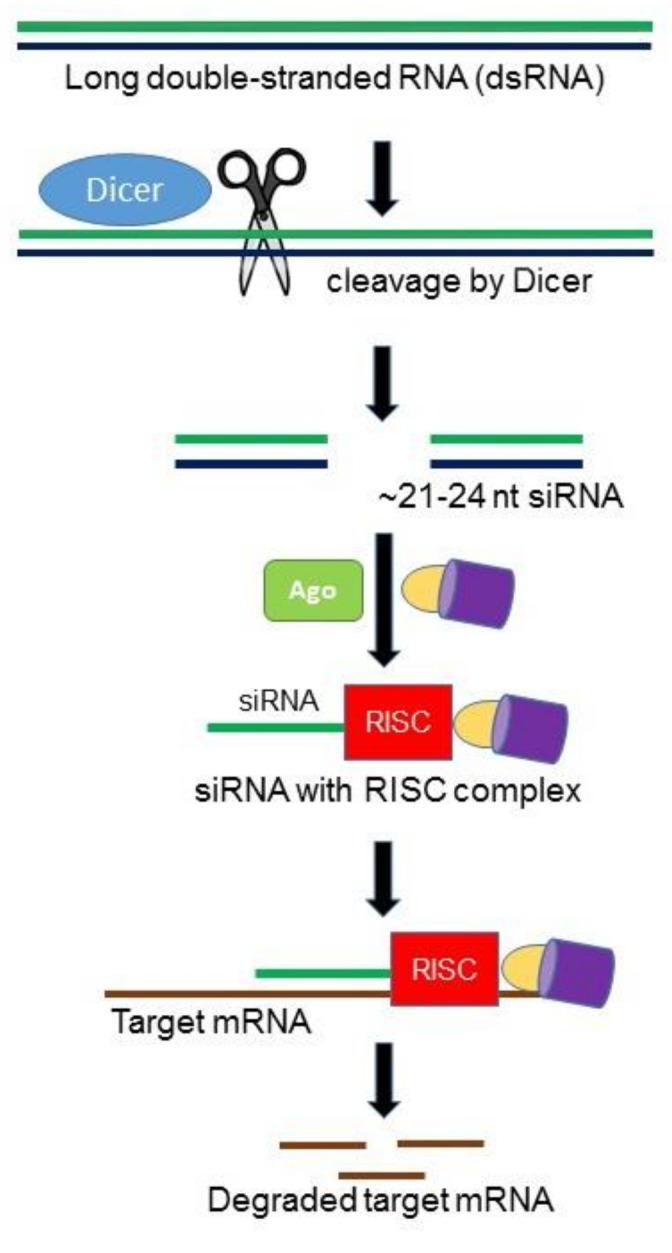
Hypothetical model of RNA interference (RNAi) pathway in fungi. The Dicer ribonuclease III enzyme (DCR) cleaves exogenous long double-stranded RNA (dsRNAs) into ~21–24 nucleotide small interfering RNAs (siRNAs). The guide siRNA then loaded onto the major catalytic component called Argonaute (Ago) and other proteins generating the RNA-induced silencing complex (RISC). siRNA, along with RISC, complementarily pair with messenger RNA (mRNA) resulting in degradation of mRNAs.

RNA interference (RNAi) is an RNA-mediated, sequence specific gene silencing mechanism involved in multiple biological processes, particularly in host defense and gene regulation [10,11]. RNAi is initiated by a RNAse III enzyme (Dicer) that cleaves a long double-stranded RNA (dsRNA) into double stranded ~21–24 nucleotides small interfering RNAs (siRNAs). Each siRNA consists of a guide strand and passenger strand. As guide strand becomes part of an active RNA-induced silencing complex (RISC), the passenger strand is degraded by the following cellular events in the cytoplasm. The guide strand of the siRNA–RISC complex base-pairs with the complementary mRNA target sequences and initiates endonucleolytic cleavage by induced Argonaute protein (AGO; catalytic component of the RISC complex), which prevents translation of the target transcript (Figure 1). Different components of the fungal RNAi machinery not only play key roles in fungal growth and development, but also important in pathogenesis. In designing a single siRNA or an RNAi hairpin construct capable of producing a number of siRNAs specific for the target gene, it is important that the siRNA(s) targeting the mRNA must have a high efficiency of silencing as well as a low probability of binding to off-target mRNAs.

Impact of RNAi on the *cyp51A* gene in the itraconazole-resistant *Aspergillus fumigatus* has shown that in addition to reducing the expression of the *cyp51A* gene, the minimum inhibitory concentration (MIC) of itraconazole has also decreased [12]. Previous studies showed that the deletion of *ERG3* and *ERG11* genes in *C. albicans* isolates induced increased azole sensitivity [13,14]. Moreover, deletion of these genes impaired the invasion of *C. albicans* into the oral mucosa [15]. Epigenetic pathways establish drug resistance in fungi by affecting a number of chromatin or RNA modifications. The changes caused by RNA are induced through small RNAs (sRNAs) and RNAi. A type of genetic mutation that contributes to resistance to rapamycin and FK506 has also been identified in *Mucor circinelloides* through RNAi pathway. Histone acetylation also activates the epigenetic machinery of chromatin. The acetylation process, which influences the nature of histone, has been shown to be one of the mechanisms of drug resistance in *C. albicans* [16,17]. Histone deacetylation-induced chromatin alteration was revealed as a function of HDAC genes which directly affects the virulence of the microorganism [18,19]. RNAi has been used as an important reverse genetics approach to understand gene function in fungi. It is inexpensive and enables us to carry out high-throughput interrogation of gene function [20,21]. However, one of the major disadvantages of RNAi is that it provides only temporary inhibition of gene function and unpredictable off-target effects [22].

### 2.2. Restriction Enzymes

Restriction enzymes are among the first generation of genome editing tools used in the field of medical mycology. These enzymes are designed to induce genome changes by cutting DNA molecules at defined points and inserting new genes at the cutting sites [23]. This mechanism of action of the restriction enzymes has made them a valuable method for cloning. However, the major issue with this method is that it is not easy to specify in advance where exactly the gene will be inserted, as the recognition sequences of most restriction enzymes are just a few base pairs long and often repeat several times in a genome. Moreover, the specificity of a restriction enzyme is dependent on environmental conditions. Since the discovery of restriction enzymes in the early 1970s, they have been widely used for the genetic manipulation of medically important fungi. Restriction enzyme mediated integration (REMI) has been employed to create mutants in medically relevant fungi including *C. albicans*, *A. nidulans*, and *A. fumigatus* [24,25]. However, it is important to note that in *C. albicans*, only heterozygous mutations are obtained. *Aspergillus and Candida* spp. are best examples of pathogenic fungi in which azole resistance mechanisms have been explored using the ability of restriction enzymes to provide a series of genetic patterns [26,27,28].

### 2.3. Zinc-Finger Nucleases (ZFNs)

Due to the mentioned limitations regarding restriction enzymes, scientists looked for ways of improving the precision of these enzymes and altering them so that they could distinguish a unique sequence in the genome. Zinc finger nucleases are examples of such unique sequences [29].

The efficiency of ZFNs as a gene editing tool is much more advanced than that of restriction enzymes. ZFN monomers are molecular proteins with two functional fused domains including the C2H2 zinc-finger (ZF) DNA-binding domain, which targets three base pairs and a non-specific catalytic domain of the FokI endonuclease. The C2H2 ZF domain is consisting of about 30 amino acids, two antiparallel β-sheets and an α-helix, and a zinc ion coordinated by two cysteine residues in the β-sheets and two histidine residues in the α-helix. FokI is a type IIS restriction enzyme involved in cleaving DNA at a distinct distance away from their recognition sites. To generate three-finger ZFs recognizing a 9-bp sequence in modular assembly, the user joins the appropriate ZF modules together. A DNA double-stranded cleavage requires dimerization of two FokI nuclease domains [30,31]. Although ZFN method was applied to gene editing in human cells and model organisms including *Arabidopsis thaliana, Caenorhabditis elegans*, and *Drosophila*, there have not been any studies done in fungi.

### 2.4. Transcription Activator-Like Effector Nucleases (TALENs)

TALENs have emerged as an alternative genome editing tool to ZFNs and are similar to ZFNs in that they can cleave their double-stranded DNA target at any desired site. Fok1 nuclease is a common functional part between ZFNs and TALENs. However, designing TALENs is more straightforward than ZNFs. In TALEN-mediated gene editing process, TALEs bind their DNA at the desired site by arrays of highly conserved 33–35 amino acid repeats that are flanked by additional TALE-derived domains at the amino-terminal and carboxy-terminal ends of the array. Each TALE repeat is largely identical, except for two highly variable residues typically found at positions 12 and 13 of the domain, referred to as the repeat variable di-residues (RVDs). This structural difference has increased the detection coefficient in TALENs and has shown higher target binding specificity as compared to ZFNs. Thus, TALENs have the ability to identify single nucleotide polymorphisms (SNPs) [32,33].

### 2.5. Clustered Regularly Interspaced Short Palindromic Repeats (CRISPR)-CRISPR Associated Protein 9 (CRISPR-Cas9)

The CRISPR (Clustered Regularly Interspaced Short Palindromic Repeats)/CRISPR-associated protein 9 (Cas9) is an adaptive immunity system in bacteria and was initially used for genome editing in mammalian and yeast cells [34,35,36]. Gradually it has become a revolutionary tool in molecular biology and biotechnology that enables us to perform precise genomic, epigenomic, RNA editing, gene expression regulation, nucleic acid detection, and several applications in a wide variety of organisms [36,37,38,39,40,41,42,43,44]. Currently, delivery of the CRISPR system’s components into fungal cells through different types of vectors and assembled purified sgRNA/Cas9 complexes have enabled us to mutate the genome or alter gene expression regulation in dozens of fungal species [36,45,46,47,48]. Alternatively, cell-penetrating peptides have been shown to be able to enter into *Candida* spp**. [49,50]**. These CRISPR-empowered enhancements have been considered a scientific breakthrough in fungal molecular biology and biotechnology [51], and stem from its versatile potential to functional characterization and breeding of clinically and industrially important fungi.

## 3. Mechanism of Action of CRISPR-Cas9

**Figure 2:**
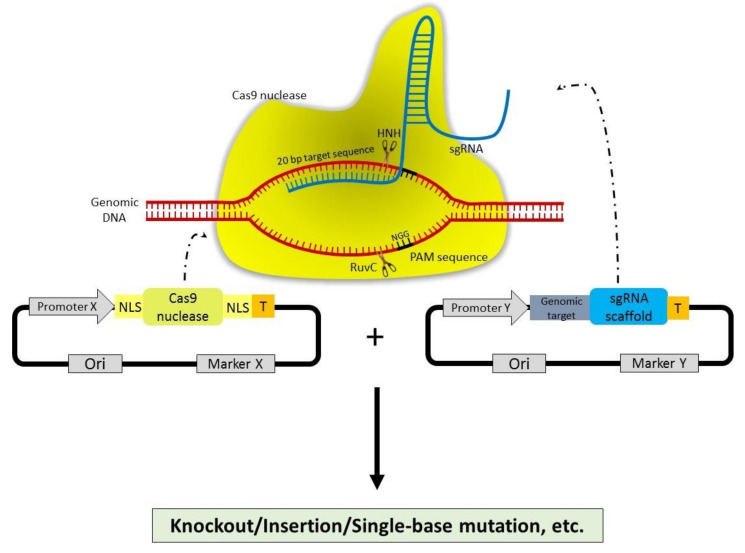
Schematic representation of RNA-guided Cas9 constructs designed for genome editing. Bottom panel: (structure of the vector plasmids used to deliver Cas9-sgRNA components into fungal cells). PromoterX can express NLS-Cas9-NLS protein. PromoterY can express 20 nt guide sequence + sgRNA cassette. Upper panel: (Cas9+sgRNA+genomic DNA). Mechanism of Cas9/gRNA ribonucleoprotein complex action, NGG (PAM site) highlighted in black line. The Cas9 nuclease domain HNH then cleaves the target DNA sequence complementary to the 20 bp guide sequence, while RuvC domain cuts another DNA strand, forming a double stranded break (DSB). DSB must be repaired via either non-homologous end joining (NHEJ) or homologous recombination (HR) immediately to avoid cell death. Insertions and deletion mutations at the target site generated by NHEJ and homology directed repair (HDR) allow disrupting or abolishing the function of a target gene. Moreover, modifications in this system can also be used to silence genes, insert new exogenous DNA, or block RNA transcription.

The type II CRISPR-Cas system (CRISPR-Cas9 from *Streptococcus pyogenes*) consists of two key molecules, including the Cas9 DNA endonuclease and a small chimeric protospacer adjacent motif (PAM)—dependent guide RNA (sgRNA). The sgRNA is composed of CRISPR RNA (crRNA) and trans-activating crRNA (tracrRNA) [45]**,** The Cas operon encodes Cas1, Cas2, and Cas9 signature proteins, and sometimes a fourth protein (Csn2 or Cas4). Moreover, repeat-spacer array alternatively contains repeats and unique spacers [52,53]. The CRISPR-array initially transcribes a precursor-CRISPR RNA (pre-crRNA), which is then processed and matured by trans-activating crRNA (tracrRNA) and RNaseIII. TracrRNA is a partial complement to a repeating sequence of crRNA, forming the chimeric sgRNA, which subsequently directs the Cas9 protein to the cleavage site. The spacer sequence of crRNA is also complementary to target DNA, such as viral nucleic acids. Then, the multifunctional and multidomain protein, Cas9, in complex with 20-nucleotide (nt) of sgRNA, cleaves both strands of target DNA preceding to protospacer adjacent motif (PAM) [54,55]. The native PAM sequence used by Cas9 is 5′NGG 3′, followed by a downstream crRNA complementary sequence that is crucial for discrimination of host and foreign nucleic acid [55,56]. The cleavage of target strand hybridizing to guide RNA and non-target strand is performed by HNH and RUVC domains of this endonuclease, respectively. Then, this fragment integrates in the CRISPR locus, allowing it to act as a new spacer sequence. Cells can detect and clear invading DNA during subsequent infection of the similar invader. This system can be effectively programmed by modifying the sequence of sgRNA to trigger desired nucleic acid cleavage. Nuclease-induced double strand breaks can be repaired by either error-prone non-homologous end joining (NHEJ) or homology directed repair (HDR) [56]. The high-fidelity HDR pathway serves as a precise gene modification by homologous recombination between donor template and repair DNA, whereas the latter leads to variable nucleotides insertion or deletion, eventually results in several mutations [56]. Figure 2 shows the schematic representation of RNA-guided Cas9 constructs designed for genome editing.

## 4. Applications of CRISPR-Cas9 Genome Editing Tool in Medically Important Fungi

### 4.1. Clinically Relevant Yeasts

*Candida* spp. are challenging for the molecular geneticist; the genome engineering and drug development against Candidiasis has been difficult for several years. Vyas and colleagues were the first to develop the CRISPR/Cas9 system in *C. albicans* [57]. Using this system, homozygous gene knock-out and multiple gene mutation have been successfully created in this fungus. Efforts to improve CRISPR-mediated genome editing have been developed in other *Candida* spp. Recently, Nguyen and colleagues reported a rapid and efficient edition without performing a molecular cloning step and utilizing permanent markers in the engineering location [58].

Since then, the CRISPR system has been utilized in several *Candida* spp. [59,60,61,62,63,64,65,66,67,68]. Therefore, CRISPR-based single and multiple gene knock-out libraries would be possible in these fungi, which further help us to develop new antifungal drugs. Cryptococcosis is a life-threatening fungal disease caused by *Cryptococcus neoformans* and *Cryptococcus gattii*. *Cryptotoccocus* spp. are found in soils contaminated with bird droppings, and in association with rotting vegetation, including eucalyptus tree hollows [69]. The prevalence of Cryptococcosis has been increasing over the past years for many reasons, including worldwide prevalence of AIDS and the expanded use of immunosuppressive drugs. Although the most common presentation of Cryptococcosis is of *C. neoformans* infection in immunocompromised people, *C. gattii* is being increasingly recognized as a pathogen in immunocompetent hosts [70]. There is an urgent need to use the CRISPR system in *Cryptococcus* spp. because low rates of homologous integration have hindered molecular genetic studies in these fungi. This limitation has been a major obstacle for the diagnosis and treatment of deadly Cryptococcal disease. Recently, the CRISPR/Cas9 system has been developed to stimulate homologous recombination (HR) for gene alteration in *C. neoformans* [71,72,73,74,75,76]. Taken together, CRISPR constructs can be successfully used for gene editing in *Cryptococcus* spp. and thus the CRISPR system would be a promising tool for drug discovery against Cryptococcosis. Strategies and applications of the CRISPR/Cas system in medically important yeasts are shown in Table 1.

### 4.2. Filamentous Fungi

Although filamentous fungi are well-known for producing high-value substances and metabolites including drugs, they have increasingly been problematic by causing life-threatening human infections [75,76,77,78,79,80]. Therefore, exploiting their genome function through applying precise and efficient techniques, thereby preventing the fungal infections, is critical. Regardless of benefit or health risks, genetic tools have not been well developed in filamentous fungi. It has been shown that polyketide synthase (PKS) is a crucial enzyme needed for toxin biosynthesis in filamentous fungi [81] and its disruption yields to engineered fungi with significant reduction in their detrimental effect to its host [82]. As a proof of principle, in a study by Fuller and colleagues, PKS in *A. fumigatus* was targeted for loss-of-function study using the CRISPR system [83]. They found high editing efficiency (25–53%) and demonstrated that the constitutive expression of Cas9 is not deleterious to *A. fumigatus* growth and other features. Additionally, Cas9-hph strain was constructed to be used as a universal recipient of sgRNA in CRISPR-based engineering. Since then, to enhance efficiency rates, in-frame integration with or without marker insertion with approximately 95–100% accuracy assisted by microhomology-mediated end joining (MMEJ) has been developed in *A. fumigatus* [84]. The genetic alteration via the CRISPR/Cas9 system has been effectively developed in *A. fumigatus* to determine the importance of various genes in *Aspergillus* [46,47,48]. For instance, the CRISPR has been applied by Umeyama and colleagues to replace *cyp51A* gene in azole-resistant clinical *A. fumigatus* isolates [46]. In this study, ribonucleoprotein complex of Cas9/gRNA and donor template have been simultaneously delivered into cells followed by testing azole susceptibility in transformants, which showed increased susceptibility via the replacement of Ser138 by glycine [46]. These studies demonstrated high performance of CRISPR/Cas9 system in *Aspergillus* research together with diversity in usability of systems components. Recently, a CRISPR-based plasmid free approach (a Cas9 RNP-mediated method) targeted for *carB* and *hmgR2* genes of *Mucor circinelloides* resulted in stable gene disruption mutants [85]. To investigate molecular pathogenesis mechanisms of *Rhizopus delemar*, single nucleotide (nt) deletions in two clinical strains FGSC-9543 and CDC-8219 were carried out [86]. Taken together, these data indicate that these approaches are simple and reliable and can be adapted in other filamentous fungi as well. Strategies and applications of CRISPR/Cas9 system in medically important filamentous fungi are shown in Table 2.

## 5. Conclusions and Future Prospects

Genetic engineering in many pathogenic fungi has been advanced in recent years and smooths the way for successful developments in the field of fungal genetics and molecular biology.

ZFNs and TALENS are useful technologies, as they facilitate researchers to create permanent mutations by introducing double-stranded breaks to activate repair pathways. However, these strategies are expensive and time-consuming to engineer, especially in large scale, high-throughput studies. A major advantage of RNAi is that a gene silencing and actual knockdown of gene expression is very simple. Unlike CRISPRi, RNAi does not target TSSs and also targets RNA transcripts in the cells’ cytoplasm and not genomic DNA in the nucleus [87,88]. However, in contrast to Cas9 and TALE systems, irreversible knockout experiments are not possible using RNAi experiment. In comparison with RNAi, studies show stronger loss-of-function phenotypes with low off-targeting effects when using CRISPRi [88]. Therefore, only from the view point of ease of use in some experiments could RNAi be ahead of CRISPRi-based technologies.

Among the various technologies, CRISPR-based techniques permit a simpler design process, and more affordable and faster execution than engineered nuclease platforms, making them highly favorable engineering tools for industrially important microorganisms with limited availability of genetic tools. The information presented in this review suggests that the CRISPR-Cas9 technique has been successfully established for several human pathogenic fungi.

For generating more than 100 mutated strains, different approaches with edition efficiencies from too low to 100% have been used, and in most cases the application of the technique consisted of a proof of concept of its feasibility. Depending on the efficiency of homologous recombination and fungal intrinsic identity, CRISPR-based genome edition may vary from a simple single gene mutation to a complex multi-gene expression regulation. Regardless of the purpose of the study, efficiency of the system might be improved through targeted change on plasmid or donor DNA [47,48,49,89]. Employment of NHEJ mutant strains was shown to be indispensable for enhancing efficiency of homologous recombination in some studies [69,90]. However, marker-less gene deletion strain construction using transient expression plasmids that can self-replicate only under antibiotic pressure is an advantage [59,91,92,93,94,95,96].

Multiplexing properties of the CRISPR system [97,98,99,100,101,102,103,104,105,106] can accelerate its ability in many aspects in the future. For instance, in development of fungal cell factories for the production of added value metabolites [107] or in development of new antifungal bioactive compounds [108], the system can be an affordable facilitator tool through approaches like genome reduction to overrule unwanted products [109] and constructing sets of deletion mutants over fungal gene interaction networks [110], respectively. A catalytically dead version of Cas9 (dCas9) has been developed by adding two point mutations in Cas9 [52], and as an extension of its application, transcriptional reprogramming [111,112] for regulation of gene expression has been introduced. These approaches might be attractive tools for gene regulation in filamentous fungi that lack components of RNAi machinery as well as in multinucleated fungi. Speed, accuracy, and sensitivity in diagnosis are critical components for cost-effective detection of fungi, subsequent prevention, and treatment of fungal infections [113,114]. Current methods of diagnosis of fungal infections are time consuming and include an expensive process of phenotypic and biochemical approaches. Thus, a demand for modern molecular diagnostic methods like genomic imaging with Cas9 has been increased. Although the CRISPR/Cas9 technique has become an increasingly important tool for several applications in line of fungal research, off-target effect, especially when using multiple sgRNAs can still limit applications of CRISPR-Cas9 system. It is worth noting that CRISPR is not the latest genome manipulation technology. Prime editing is the newest class of known gene editing methods that has been introduced in the field of biotechnology since 2019. Prime editing, which can be used in biological systems, first searches for the points in the gene that need to be modified, then impose the editing effects, without breaking into double stranded DNA at the target points, through base deletion, insertion, and substitutions. The most notable advantage of this method to CRISPR is that it has less off-target effects and is more precise [115,116,117]. This technique is still in its infancy and has not yet been used in pathogenic fungi.

## Figures and Tables

**Table 1:** Modules, applications and success rate of the CRISPR/Cas9 system in manipulating clinically relevant yeasts.

Organism	CAS9 Expression Module	GRNA Expression Module	Target Gene (S)	Purpose of Application	Editing Rate and Result	References
***C. albicans***	*Candida/Saccharomyces* codon–optimized version of Cas9 (CaCas9)/the ENO1 promoter	The RNA polymerase III (Pol III) promoter SNR52	*ADE2, CDR1/CDR2*	To generate homozygous mutations in one transformation by Duet and Solo system	Duet system showed 20–40% mutagenesis efficiency, and Solo system enabled 60–80% targeting	Vyas et al. (2015) [57]
***C. albicans***	Transient CRISPR-Cas9 system by using a SAT1-FLP system	SNR52P/TENO1	NDT80, REP1, and RON1	To better understand role of target genes (single or in combination) in virulence	Single, double, and triple deletion strains were successfully constructed	Min et al. (2018) [60]
***C. albicans***	US-pENO1 ˃ Cas9-NAT	NAT-pSNR52-gRNA-DS	ADE2, URA3, WOR1,WOR, and CZF1	To develop a marker less system without need for molecular cloning step	80% single gene deletion, 20% double genes deletion and ˃50% integration efficiency	Nguyen et al. (2017) [58]
***C. albicans***	CIp-ARG4-PTEF CaCAS9	*PADH1-tRNA*-driven gRNA expression	*RFP*	To optimize gRNA intracellular expression	Increase the gene editing efficiency by 10-fold	Ng et al. (2017) [64]
***C. albicans***	CaCas9 into the *C. albicans* genome at the *NEUT5L* locus	5′ homology arm–SNR52 promoter–gRNA1–gRNA2-3′ homology arm	antifungal efflux and biofilm adhesion factors	To develop a gene drive array system for the generation of combinatorial deletion mutants	Two larges pairwise gene deletion mutants were successfully generated	Shapiro et al. (2018) [63]
***C. albicans***	the ENO1 promoter/Cas9 (CaCas9)/T_CYC1_	SNR52P/T_ENO1_	*ADE2*	To describe a transient CRISPR-Cas9 system for efficient gene deletion	Homozygous deletions by introduction of CaCas9 transiently	Min et al. (2016) [59]
***C. parapsilosis***	TEF1p-CAS9-TEF1t	pCpSNR52-sgRNA-SUP4t and cpGAPDHp-HH-sgRNA-HDV-GAPDHt	*ADE2*, *CPAR2_101060 and URA3*	To apply gene manipulation in single transformation step which can be used for editing of any number of target genes	The system yielded up to 100% efficiency across a panel of 20 clinical isolates	Lombardi et al. (2017) [66]
***C. glabrata***	pTEF1-Cas9-tCYC1/pCYC1-Cas9-tCYC1	pSNR52-sgRNA-tTY2/pRNAH1-sgRNA-tTY2	*ADE2, VPK1 and YPS11*	To establish a loss-of-function mutation through the NHEJ repair pathway	High	Enkler et al. (2016) [61]
***C. glabrata***	pTEF-Cas9-KanMX	p*SNR52*-sgRNA-*CYC1*t	*ADE2*, *MET15* and *SOK2*	To compare genome modifications in *C. glabrata* wild type and *lig4* strains	Targeting efficiency in the *lig4Δ* mutant was higher than in the wild type strain	Cen et al. (2017) [62]
***C. albicans***	Codon-optimized version of Cas9(CaCas9)-SV40NLS	SNR52 RNA polymerase III promoter	*CDR1 and CDR2*	To present a modified gene-drive-based assay for gene manipulation	−	Halder et al. (2019) [65]
***C. albicans***	ACT1p-dCAS9-ACT1t	SNR52p-gRNA tail	*ADE2*	To demonstrate a functional CRISPRi system for transcriptional repression	20-fold repression of target gene achieved	Wensing et al. (2019) [68]
***C. parapsilosis, C. orthopsilosis, C. metapsilosis and C. tropicalis***	MgTEF1p-CAS9-MgTRP1t	pAgTEF1-sgRNA-HDV-ScCYC1t	*ADE2 and CPAR2_101060*	To construct an autonomously replicating plasmid for markerless ediing in *Candida* spp.	Single gene distribution efficiency observed in *C. parapsilosis* (approximately 80*%*)*, C. meta psilosis* (100%), *C. tropicalis* (88–100%)	Lombardi et al. (2019) [67]
***Cryptococcus neoformance***	TEF1p-Cas9-SV40NLS-TEF1t	pACT1-HH-gRNA-HDV-TRPt	*ADE2*	To demonstrate the first proof of principle study	70%	Arras et al. (2016) [71]
***C. neoformans***	ACT1P-SV40NLS-Cas9-NLS-bGHpAt	pCnU6-GN19-gRNA-6Ts	*ADE2* and *Tsp2-1*	To develop a system for gene alterations by subsequent complementation and off-target effects reduction	Frequency of gene deletion was over 80%, indel efficiency and HR rates were 40–90% and 20–90%, respectively	Wang et al. (2016) [72]
***C. neoformans***	GPD1p–Cas9-GPD1 t	pCnU6-sgRNA-6-Tt	*ADE2*	To generate a TRACE system as an cost-effective and efficient strategy for genetic modifications	Up to 90% gene disruption rate	Fan et al. (2018) [73]
***C. neoformans***	pTEF-Cas9-FLAG-NLS	ptRNA-sgRNA-NLS	*GIB2*	To deliver a preassembled RNP via electroporation to accelerate of gene editing	Approach is sufficient to induce gene modification	Wang P. (2018) [74]

**Table 2:** Modules, applications and success rate of the CRISPR system in manipulating clinically relevant molds.

Organism	CAS9 Expression Module	GRNA Expression Module	Target Gene (S)	Purpose of Application	Editing Rate and Result	References
***A. fumigatus***	p-hph-Ptef1-cas9	p426-SNR52p-gRNA.CAN1.Y-SUP4t	*PKSP*	To test CRISPR-CAS9 method in this organism	High gene targeting efficiency reached 25–53%	Fuller et al. (2015) [83]
***A. fumigatus***	Gpdap-3xFLAG-NLS-Cas9-NLS-TRPCt	U6-3-gRNA	*pksP* and *cnaA genes*	To establish the system for mutagenesis using MMEJ process	Approximately 95–100% rate of mutagenesis obtained	Zhang et al. (2016) [84]
***A. fumigatus***	Alt-R-CRISPR-Cas9 components from integrated DNA technologies (IDT)	cr5 = pksP and cr3 = pksP	*PKSP*	An in vitro assembly of RNP demonstrated to eliminate the strain construction step	Gene deletion efficiency was close to 100%	Al-Abdallah et al. (2017) [48]
***A. fumigatus***	Cas9-NLS	T7-sgRNA	*CYP51A*	To investigate the mechanisms of azole resistance via cyp51A alteration	Site-directed mutagenesis successfully performed using CRISPR-CAS9 system	Umeyama et al. (2018) [46]
***Mucor circinelloides***	Alt-R CRISPR-Cas9 tracrRNA	Alt-R CRISPR crRNA	*CARB* and *HMGR2*	To obtain mitotically stable mutants, a plasmid free CRISPR-Cas9 approach demonstrated	Targeting efficiency of NHEJ and HR reach to 100%	Nagy et al. (2017) [85]
***Rhizopus delemar***	pmCas9: tRNA-gRNA	pmCas9: tRNA-gRNA	*PYRF*	For investigating molecular pathogenesis mechanisms, point mutation introduced	Efficiency of 36% to 59%	Bruni et al. (2019) [86]

## Data Availability

Not applicable.

## Abbreviations

Ago	Argonaute
AIDS	acquired immunodeficiency syndrome
CRISPR	clustered regularly interspaced short palindromic repeats
CRISPR-Cas9	clustered regularly interspaced short palindromic repeats associated protein 9
Cas9-NLS	cas9 with a nuclear localization signal
crRNA	CRISPR RNA
dCas9	dead version of Cas9
DSB	double stranded break
dsRNA	double-stranded RNA
HR	homologous recombination
HDR	homology directed repair
IDT	integrated DNA technologies
MIC	minimum inhibitory concentration
mRNA	messenger RNA
NHEJ	non-homologous end joining
NGG	nucleotide- guanine-guanine
PAM	protospacer adjacent motif
PKS	polyketide synthase
REMI	restriction enzyme mediated integration
RISC	RNA- induced silencing complex
RVD	repeat variable di-residues
SAT1-FLP	SAT1 flipper
siRNA	small interfering RNA
sgRNA	single guide RNA
SNP	single nucleotide polymorphism
TALEN	transcription activator-like effector nucleases
tracrRNA	trans-activating crRNA
ZFN	zinc-finger nuclease
